# Pluripotent Stem Cells in Clinical Cell Transplantation: Focusing on Induced Pluripotent Stem Cell-Derived RPE Cell Therapy in Age-Related Macular Degeneration

**DOI:** 10.3390/ijms232213794

**Published:** 2022-11-09

**Authors:** Yi-Ping Yang, Yu-Jer Hsiao, Kao-Jung Chang, Shania Foustine, Yu-Ling Ko, Yi-Ching Tsai, Hsiao-Yun Tai, Yu-Chieh Ko, Shih-Hwa Chiou, Tai-Chi Lin, Shih-Jen Chen, Yueh Chien, De-Kuang Hwang

**Affiliations:** 1 Department of Medical Research, Taipei Veterans General Hospital, Taipei 112201, Taiwan; 2 Institute of Pharmacology, College of Medicine, National Yang Ming Chiao Tung University, Taipei 112304, Taiwan; 3 School of Medicine, National Yang Ming Chiao Tung University, Taipei 112304, Taiwan; 4 Institute of Clinical Medicine, National Yang Ming Chiao Tung University, Taipei 112304, Taiwan; 5 Department of Ophthalmology, Taipei Veterans General Hospital, Taipei 112201, Taiwan; 6 Genomics Research Center, Academia Sinica, Taipei 115201, Taiwan

**Keywords:** pluripotent stem cells, embryonic stem cells, induced pluripotent stem cell, clinical trials, retinal pigment epithelial cells, age-related macular degeneration, cell transplantation

## Abstract

Human pluripotent stem cells (PSCs), including both embryonic stem cells (ESCs) and induced pluripotent stem cells (iPSCs), represent valuable cell sources to replace diseased or injured tissues in regenerative medicine. iPSCs exhibit the potential for indefinite self-renewal and differentiation into various cell types and can be reprogrammed from somatic tissue that can be easily obtained, paving the way for cell therapy, regenerative medicine, and personalized medicine. Cell therapies using various iPSC-derived cell types are now evolving rapidly for the treatment of clinical diseases, including Parkinson’s disease, hematological diseases, cardiomyopathy, osteoarthritis, and retinal diseases. Since the first interventional clinical trial with autologous iPSC-derived retinal pigment epithelial cells (RPEs) for the treatment of age-related macular degeneration (AMD) was accomplished in Japan, several preclinical trials using iPSC suspensions or monolayers have been launched, or are ongoing or completed. The evolution and generation of human leukocyte antigen (HLA)-universal iPSCs may facilitate the clinical application of iPSC-based therapies. Thus, iPSCs hold great promise in the treatment of multiple retinal diseases. The efficacy and adverse effects of iPSC-based retinal therapies should be carefully assessed in ongoing and further clinical trials.

## 1. Introduction

Owing to two properties (self-renewal and potency), major advances in the therapeutic effects of cell therapy have been made in pluripotent stem cells (PSCs) in stem cell research. Self-renewal is the capacity of stem cells to divide indefinitely and produce daughter cells that maintain the same properties as the progenitor cells. Under specific conditions of pluripotency-associated factors, a stem cell ceases self-renewal and enters a differentiation program that develops into the three primary groups of cells that make up a human body, or the three germ layers (ectoderm, endoderm, and mesoderm)**.** Relying on the differentiation potential of PSCs, PSCs can be differentiated into different retinal lineage cells, including retinal pigment epithelium cells (RPEs) and retinal ganglion cells (RGCs), which may show therapeutic effects in age-related macular degeneration (AMD) and glaucoma, respectively. It was reported that transplanted primary RGCs showed responses to light in rat retinas [1]. The transplantation of stem cells derived from bone marrow [2,3] and human periodontal ligament [4] also alleviated disease manifestations in rodent models of RGC degeneration, probably due to the neuroprotective effects of these stem cells. Remarkably, the transplantation of PSCs such as embryonic stem cells (ESCs) improved visual acuity in mice with drug-induced RGC deletion [5]. Recently, we optimized transplantation strategies and transplanted ESC-derived RGCs into recipient mice in which the transplanted PSC-derived RGCs integrated and formed synapses [6]. However, as a part of the central nervous system (CNS) that can transmit visual information from the retina to the primary visual cortex, the optic nerve is generally unable to regenerate or be repaired. Despite the progress of the aforementioned studies using RGC transplantation, the replacement or repair of the damaged optic nerve remains a difficult issue in clinical application. On the other hand, RPEs, which form the single-layer epithelium outside the neurosensory retina, play crucial roles in the transduction of normal visual functions. To reverse the RPE loss, replace damaged RPEs, or restore RPE physiology in progressive AMD, several attempts and efforts have been made in many in vitro studies, animal studies, preclinical studies, and many clinical trials. In this review article, we conducted a literature review to evaluate past and ongoing ESC- and iPSC-based clinical trials related to various diseases, with a particular focus on PSC-based cell therapy for age-related macular degeneration (AMD). This review aims to highlight current knowledge on the potential and feasibility of advanced PSC therapeutics in the treatment of AMD.

## 2. Updates on Cell Therapy and PSC-Based Clinical Trials

**Figure 1:**
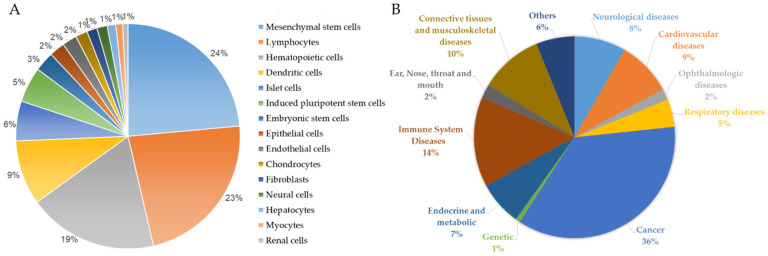
Frequency distribution of recruiting, completed, and enrolling cell-based clinical trials in the past decade. (**A**) Pie chart showing the relative frequency distribution of clinical trials using indicated cell type. (**B**) Pie chart showing the relative frequency distribution of clinical trials for indicated diseases (from URL https://clinicaltrials.gov/, accessed on 16 August 2022).

Recent cell therapy-based clinical studies conducted by various research groups showed the role of stem cells in replacing damaged tissues and promoting endogenous cellular regeneration for various diseases (Figure 1). Several cell types, including primary cells and various stem cells, have been employed in this field. Clinical trials using PSCs are on the rise based on their dynamic niche in regenerative medicine. Two commonly used types of PSCs are ESCs and induced pluripotent stem cells (iPSCs) [7]. ESCs are isolated from the inner cell mass of preimplantation embryos at the blastocyst stage. iPSCs, on the other hand, are obtained via the in vitro reprogramming of human-derived somatic cells back to their embryonic-like pluripotent state. Both ESCs and iPSCs are capable of proliferating indefinitely and differentiating into all the derivatives of the three germ layers. Cell therapy using a broad range of iPSC-derived cell types is now evolving rapidly, especially in Japan and the USA. Furthermore, iPSC-derived cell types and their application include iPSC-derived mesenchymal stem cells (MSCs) for steroid-resistant graft-versus-host disease (GVHD), iPSC-derived dopaminergic progenitors for Parkinson’s disease, iPSC-derived cardiac progenitors for heart failure iPSC-derived platelets, iPSC-derived beta-pancreatic cells for type I diabetes, iPSC-derived natural killer cells (NK cells) for advanced solid tumors, and iPSC-derived RPEs for retinal disorders.

**Figure 2:**
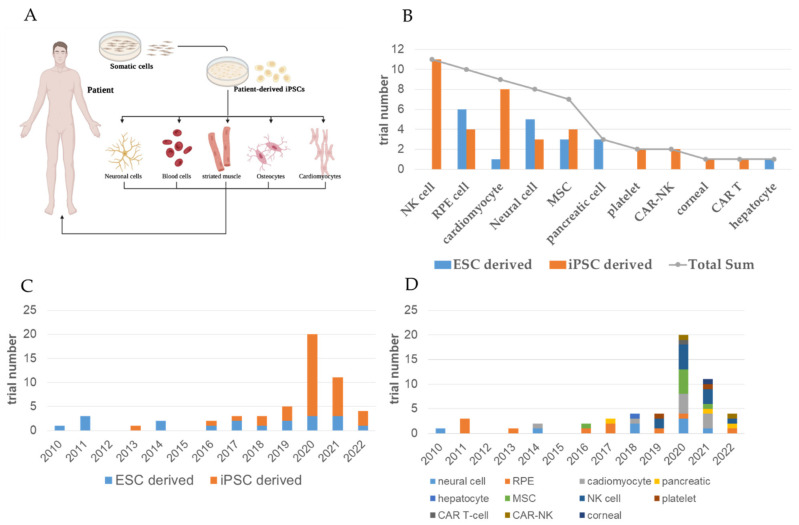
Recruiting and completed interventional clinical trials of approved iPSC- and ESC-based treatments. (**A**) Scheme depicting the generation of patient-derived iPSCs and their potential to differentiate into various cell lineages. (**B**) Bar chart showing the number of recruiting and completed interventional clinical trials using indicated cell type. (**C**) Bar chart showing the number distribution of clinical trials over years Treatment type distribution of over the years. (**D**) Bar chart showing the number distribution number of recruiting and completed interventional clinical trials using indicated cell type over the years. (Panel (**A**): Created with BioRender.com).

Stem cell-based cell transplantation is not a new concept in the hematological research community. The use of hematopoietic stem cells (HSCs) to reconstitute malfunctioning blood systems has been widely adopted over the last four decades. However, there were concerns over its success rate, and scientists began looking for alternatives to circumvent the limitations posed by the scarce number of HSCs available for transplantation, which gave rise to the development of HSCs and blood cells using iPSC technologies [8]. Sawa and Miyagawa et al. reported the world’s first case of ischemic cardiomyopathy, whereby the patient received a transplantation of iPSC-derived cardiomyocyte (iPSC-CM) patches and showed an improved prognosis (#jRCT2053190081) [9]. In 2018, Professor Takahashi used clinical-grade iPSCs to produce dopaminergic neurons and transplanted them into Parkinson’s disease patients (#JMA-IIA00384) [10]. Professors Okano and Nakamura et al. generated iPSC-derived neural stem/progenitor cells and transplanted them into patients with subacute spinal cord injury in 2019 (#jRCTa031190228) [11]. In 2021, Professor Fujita and Kishino et al. developed human iPSC-derived cardiac spheroids and used them to improve cardiac function in animal models with heart failure [12]. They also assessed the utilities of human iPSC-derived cardiac spheroids in patients with severe heart failure in Phase I/II clinical trials (#jRCTa032200189) [13] (Figure 2).

## 3. From ESC to iPSC: Past and Ongoing iPSC- and ESC-Based Clinical Trials

First discovered in 1998, ESCs have been extensively explored in pre-clinical and clinical studies, even until the recognition of iPSCs in 2006 and the generation of the first human-derived iPSCs by Nobel laureate Shinya Yamanaka in 2007 [14]. ESCs and iPSCs have been widely used for cell transplantation, particularly in the retina. A team led by Professor Robert Lanza from the USA explored the potential of subretinal hESC-derived retinal pigment epithelial cell (hESC-RPE) transplantation. Preclinical studies demonstrated that the transplantation of hESC-derived RPEs led to long-term improvements in visual function (>220 days) in experimental animal models of macular degeneration and Stargardt disease. After transplantation, the hESC-derived RPEs improved photoreceptor and visual function in a dose-dependent manner without tumorigenic incidence [15]. hESC-derived RPEs have since been subjected to transplantation into human patients with macular degeneration in a preliminary report [16] and two prospective phase 1/2 studies [17]. No signs of hyperproliferation, tumor formation, graft rejection, or other safety issues were observed 4 months [16] and 12 months after transplantation [17]. A team led by Professor Peter Coffey reported the successful delivery and survival of an hESC-derived RPE monolayer on a coated, synthetic basement membrane in patients suffering from severe wet AMD. Twelve months after the transplantation, visual acuity was improved by 29 and 21 letters in the two patients, respectively [18]. On the other hand, Kashani et al. reported a phase 1/2a clinical trial involving the subretinal transplantation of polarized hESC-derived RPEs on an ultra-thin parylene substrate in advanced dry AMD [19]. One year post-transplantation, the subretinal implants were generally safe and tolerated by these patients [19]. The first successful attempt at subretinal transplantation of iPSC-derived RPEs was made by Professor Masayo Takahashi in Japan in 2014 (#UMIN000011929). Considering the use of immunosuppressants in transplantation using hESC-derived RPEs, Takahashi et al. generated patient-specific iPSC-derived RPEs from an elderly patient with severe AMD and conducted subretinal transplantation in the patient without the need for immunosuppressants [20]. After a four-year follow-up, the transplanted patient-specific iPSC-derived RPEs still appeared functional and showed no immunological issue without the use of immunosuppressants [21].

**Figure 3:**
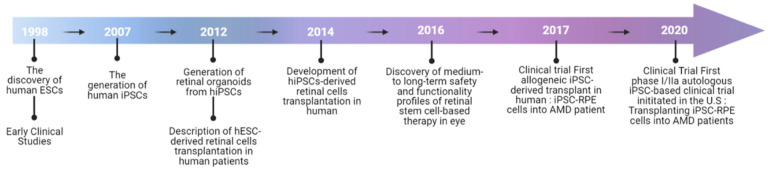
Timeline of the application of PSC-derived retinal cells in clinical studies.

Retinal organoids were first generated from these hiPSCs in 2012, which later led to the first successful transplantation of hiPSC-derived retinal lineages in humans in 2014 [20] (Figure 3). The beginning of iPSC- and ESC-based therapies started with the eye, which is said to be an ideal organ because of the optical and surgical accessibility of its internal structures and the increase in the already numerous noninvasive treatments. The preparation of preclinical testing in stem cell-based therapy includes cell sourcing, the manufacturing of clinical-grade cells, and preclinical animal studies. Many comparisons have been made between iPSCs and ESCs; however, both cells still have their own advantages and disadvantages. Although ESCs have been extensively studied and are immunologically more compatible with the host, iPSCs are easier to culture, and there is no ethical concern attached to such PSCs. Studies have shown that iPSCs can be generated from somatic cells isolated from various tissues such as skin, dental tissue, blood, and urine [22,23]. Therefore, iPSCs have shown fewer ethical problems compared to ESCs. Furthermore, iPSCs offer advantages and have many biomedical applications such as drug screening, toxicological studies, disease modeling, and cell therapy.

## 4. RPE Physiology and Therapeutic Options for AMD

**Figure 4:**
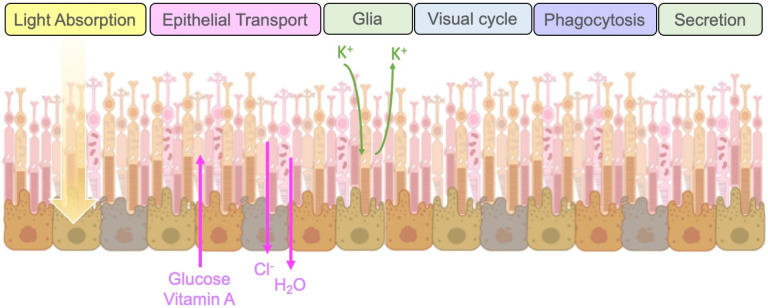
General physiological functions of RPEs. The general functions of RPEs include light absorption, the trans-epithelial transport of molecules and ions, the spatial buffering of ions, regulation of the visual cycle, phagocytosis of shed photoreceptor membranes, and the secretion of growth factors. (Image credit—Created with BioRender.com).

The RPE is a single-layer epithelium under the neurosensory retina that rests on the basal Bruch’s membrane and is essential for normal visual transduction. In addition to vision, it plays multiple roles, including the provision of the outer blood–retina barrier and fluid transport between the choroid and the neural retina, the regulation of cytokine release, the processing of reactive oxygen species, the recycling of phototransduction components, and the regulation of ionic balance in the subretinal space. RPE cells act as metabolic sensors; they influence the vascular tone and photoreceptor function [24] by balancing the secretion of trophic growth factors such as vascular endothelial growth factor (VEGF), a major angiogenic stimulator, and pigment epithelium-derived factor (PEDF), a potent angiogenic inhibitor. Additionally, RPE cells secrete neurotrophic factors such as GDNF, CNTF, and BDNF, and synthesize dopamine, suggesting potential therapeutic effects of RPE cell transplantation in Parkinson’s disease [25]. In normal eyes, the apical secretion of PEDF into the interphotoreceptor matrix provides neurotrophic activities on photoreceptors, while the basal secretion of VEGF supports the maintenance of the choriocapillaris [26]. Therefore, stabilizing the intraretinal vascular networks by keeping the RPE secretion of trophic growth factors under control is key to preventing vision loss [27]. The general functions of RPEs include light absorption, the transepithelial transport of molecules and ions, the spatial buffering of ions, regulation of the visual cycle, phagocytosis of shed photoreceptor membranes, and the secretion of a variety of growth factors [24], and are summarized in Figure 4.

AMD is the leading cause of severe central visual impairment in older populations in developed countries. The diagnosis of AMD is achieved using imaging modalities, including optical coherence tomography (OCT), fluorescein angiography (FA), and indocyanine green angiography (ICGA). If left untreated, an irreversible disciform scar will form, destroy the neural architecture of the macula, and lead to loss of vision in the central visual field. The current treatments for AMD mainly involve the intravitreal injection of anti-vascular endothelial growth factor (anti-VEGF) drugs, such as bevacizumab, ranibizumab, and aflibercept [28,29]. Anti-VEGF agents are injected monthly or bimonthly and are effective for recurrent neovascularization on a pro re nata basis, especially for individuals with high baseline visual acuity or those who began treatment earlier. However, anti-VEGF agents do not target the underlying degeneration inherent in the disease, and the need for regular follow-up intravitreal injections imposes a heavy economic and physical burden on patients. The current treatments for wet AMD are summarized in Table 1. There is currently no treatment for the dry type of AMD. Before the widespread use of anti-VEGF agents, several other treatment modalities were used. This included laser photocoagulation to ablate fovea-sparing CNV lesions, submacular surgery to remove the neovascular membrane, and photodynamic therapy (PDT) to treat choroidal tissues by injecting the photosensitizing drug verteporfin. While laser photocoagulation destroys the overlying retinal tissue and is damaging to foveal lesions, submacular surgery has shown no benefits in cataract progression and retinal detachment, and PDT still results in vision loss in most patients [30]. This has led to a call for other surgical approaches for patients, such as macular translocation surgery and mechanical displacement of the subretinal hemorrhage using gas; however, there is still insufficient evidence to recommend macular translocation to patients. Therefore, it is plausible that devising a method to regenerate RPE cells, improve their function, and prevent continued aging is an important foundation for treating AMD.

Innovative therapeutics for AMD were initially developed in preclinical animal studies. Various experimental models such as transgenic mice, OXYS rats, rabbits, and nonhuman primates have been used to recapitulate RPE dysfunction for modeling AMD [31,32,33,34]. It has been shown that small molecules and cell transplantation both exhibit potential in the treatment of AMD. Small molecules such as ABCF1, melanin, LEDGF, and ramoplanin have been developed and used to restore RPE function in phagocytosis and metabolic activity [35,36,37,38,39]. However, relatively short half-lives of small molecules have also been observed, which may hinder the application of small molecules. The clinical translation of these small-molecule drugs is even more challenging due to the uncertainty of pharmacokinetic and pharmacogenetic conditions. Compared to small molecules, cell transplantation-based cell therapies are a rather a straightforward approach that can encourage cell repopulation and the restoration of organ function. Therefore, cell transplantation using iPSCs has been increasingly applied for the treatment of AMD in experimental models [15,40,41].

## 5. Application of iPSC-Derived RPEs in Disease Modeling and Cell Therapy

**Figure 5:**
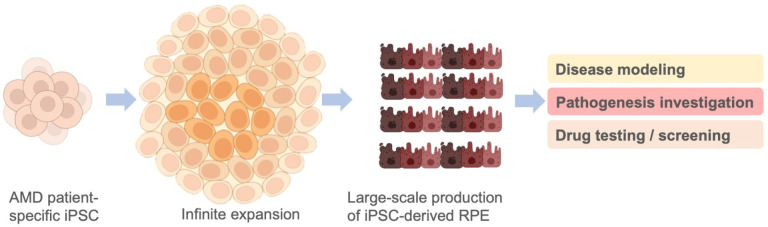
Potential of patient-specific iPSC-derived RPEs for large-scale production. Patient-derived iPSCs can be easily obtained from patients’ somatic cells and expanded infinitely in culture, allowing the large-scale production of iPSC-derived RPEs to meet the demand of disease modeling, pathogenic mechanism investigation, and drug testing/screening. (Image credit—Created with BioRender.com).

Both the primary culture of RPEs from human adult donors and iPSC-derived RPEs are in vitro human RPE cell models and have been utilized as in vitro systems for the disease modeling of AMD and for the testing of treatment response [42]. However, the primary human adult RPEs that need to be isolated from deceased human donors carry several limitations, including the donor tissue’s low availability and the poor ability of such RPEs to expand in culture. These disadvantages largely have hindered the bioavailability of primary human adult RPEs as in vitro models for pathogenic mechanism investigations and for the screening of potential therapeutics. Compared with primary human adult RPEs, iPSC-derived RPEs can be generated from somatic cell sources, which can be easily obtained. In addition, iPSCs can expand infinitely, allowing iPSC-derived RPEs to be produced in large amounts. Several reports have indicated impaired metabolism and dysregulated gene expression in patient-specific iPSC-derived RPEs from AMD patients, as well as the feasibility of using iPSC-derived RPEs to investigate AMD pathogenic mechanisms [43,44,45,46,47,48] (Figure 5). In addition to primary human adult RPEs and iPSC-derived RPEs, some advanced in vitro cell models, such as human stem cell-derived 3D organoid systems [49,50,51,52], subretinal chips [51,52,53], and bioprinting platforms [49], have also been used to improve the efficiency and therapeutic translatability of preclinical studies and drug screening. The multi-modal chip also sets up a novel platform of bio-subretinal prostheses that are implantable and may have the potential to restore vision [53].

**Figure 6:**
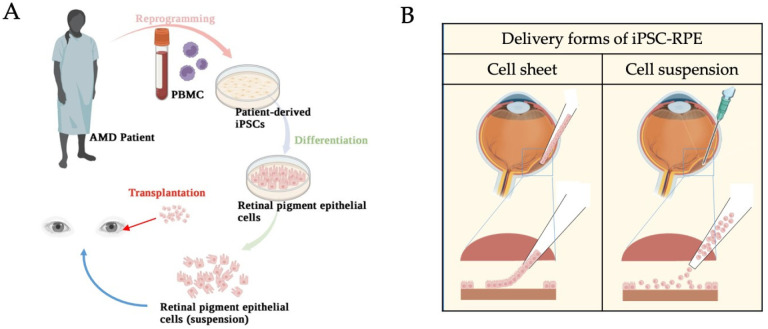
Schematic diagrams of iPSC-RPE transplantation. (**A**) Peripheral blood mononuclear cells (PBMCs) from the AMD patient were reprogrammed into iPSCs, followed by differentiation into iPSC-derived RPEs. The iPSC-derived RPE cells, as cell suspension, were transplanted back into the patient’s subretinal space. (**B**) Delivery forms of transplantation in the eye. (Image credit—Created with BioRender.com).

The development of iPSC technologies made in vitro studies of pluripotent differentiation and tissue transplantations possible. During AMD progression, it has been widely indicated that RPE is the key target for AMD treatment. The purpose of RPE transplantation in the treatment of AMD is to replace RPE cells and support overlying photoreceptors and surrounding tissues, such as the underlying choriocapillaris. Overall, the delivery of stem cell-derived RPE has been primarily implemented in two forms, namely, RPE cell suspensions and an RPE monolayer with scaffold support (Figure 6). Significant advances are being made in the development and delivery of each form. Cell suspensions are generally cryopreserved indefinitely until the time of surgery, when they are thawed, washed, and then, resuspended in a buffer solution. The density and volume are amenable to subretinal injection and the final target dose of RPE cells in suspension ranges from ~50,000 to 200,000 (ClinicalTrials.gov, NCT01469832) [16,17]. The delivery of these RPE cells could be intravitreal via a small-gauge cannula, or suprachoroidal. On the other hand, cell monolayer sheets demand greater complexity in the manufacturing process as they require a scaffold to support the cells. The scaffold-supported monolayers are more complex and expensive but better mimic the morphology and characteristics of the endogenous RPE cells. The surgical delivery of cell sheets has demonstrated effective targeting of geographic atrophy (GA) (ClinicalTrials.gov, NCT02590692) [54]. A range of recruiting, enrolling, and completed PSC (iPSC and ESC)-based clinical trials related to retinal cells are documented by ClinicalTrials.gov (summarized in Table 2). Considerations of tumorigenicity, immunogenicity, barrier function, maturity, and viability have been at the center of clinical trials for both RPE cell suspension and sheet formulations. Although no significant visual loss has been reposted in subjects using any approach, polarized RPE cell sheets showed higher resistance to oxidative insult-induced cell death than non-polarized RPEs, owing to their constitutively higher expression of antioxidants and cell survival signaling, and lower levels of proapoptotic signaling pathways [55].

The use of immunosuppressants was indicated as a major adverse effect in clinical trials using ESC-derived RPE cell suspension [16,17,56]. Therefore, Takahashi et al., at the Riken Center in Japan, tested the feasibility of autologous iPSC-derived RPE cell sheets for transplantation [57,58], and subsequently published the first case of autologous iPSC-RPE transplantation [20]. The patient-derived iPSC-RPEs, generated from an elderly patient with severe AMD, were extensively tested and the resultant iPSC-RPE cell sheets were used for subretinal transplantation in the AMD patient [20]. The 1-year follow-up results of this patient showed that the transplanted iPSC-derived RPE cell sheet remained intact and the post-operative best-corrected visual acuity was maintained, but not improved, without the use of immunosuppressants [20]. At the 4-year follow-up, multimodal imaging confirmed that the transplanted iPSC-derived RPE cell sheet was still viable and simultaneously supported the photoreceptors and choroidal vasculature [21]. These findings indicated that this autologous iPSC-derived RPE transplantation for AMD treatment is safe and feasible [20,21]. Early-phase iPSC- and hESC-based clinical trials have shown preliminary signs of efficacy and safety and have highlighted surmountable challenges, such as the immunological selection criteria for the use of cell suspensions versus RPE sheets [59]. Therefore, more pre-clinical and clinical efforts are needed to expound the potential of stem cell-derived RPE in AMD treatment.

## 6. Conclusions

Stem cells have been widely used in various clinical studies for replacing damaged tissues and promoting endogenous cellular regeneration in various diseases. PSC lines have exhibited promising advantages, although several questions on the optimization of cell resources remain, including the costs, the availability of starting materials, and the risk of immune system rejections during transplantation. To overcome the problem of immune system compatibility, researchers have explored the idea of matching HLA (human leukocyte antigen) haplotype profiles between donors and patients, making their cells or tissues more compatible with unrelated patients. HLA-matched allogeneic transplantations can eliminate the need for immune suppression and are safer for the patients [60,61].

In conclusion, stem cell-based therapeutic strategies using adult somatic stem cells, and hESC- and iPSC-derived RPEs, demonstrate promising potential, and early-phase clinical trials have shown preliminary signs of efficacy and safety. This review provides a comprehensive evaluation of past and ongoing stem cell-based treatments related to various diseases over the past two decades worldwide. Taken together, more pre-clinical and clinical efforts are anticipated to expound the potential of stem cell-derived tissue transplantations.

## Figures and Tables

**Table 1:** Current Treatments for wet AMD.

Treatments	Formulation	Procedure	Administration	Frequency	Main Function
Anti-VEGF therapy	Antiangiogenic drugs	Intravitreal injection, infusion	Monthly/weekly	More than 1	Blocking or neutralizing VEGF expression
Laser photocoagulation	Laser light with special contact lens	Laser surgery	Monthly	More than 1	Utilizing heat from a laser to shrink or destroy abnormal blood vessels
Photodynamic combined therapy	Laser- and light-activated drugs	Intravenous injection and shining a laser into the eye	Monthly	More than 1	Creating blood clots to seal the abnormal blood vessels
Cell therapy	Pluripotent stem cell-derived RPE cells	Subretinal injection or transplantation	Once	1	Reversal of the degenerative loss of RPE cells

**Table 2:** Interventional clinical trials of ESC- and iPSC-based studies in retinal therapy with recruiting, enrolling via invitation, and completed status.

	Cell	Trial ID	Trial Title	Condition	Status	Region	Age	Sample	Sponsors	Trial	Transplant	Phase	Delivery
1	ESC-RPE	NCT03167203	A Safety Surveillance Study in Subjects with Macular Degenerative Disease Treated with Human Embryonic Stem Cell-derived Retinal Pigment Epithelial Cell Therapy	MD	Enrolling via invitation	UK, US	18 years and older	36	Astellas Institute for Regenerative Medicine	Allogenic	Suspension	I/II	Subretinal injection
2	ESC-RPE	NCT03963154	Interventional Study of Implantation of hESC-derived RPEs in Patients with RP due to Monogenic Mutation	RP	Recruiting	France	18 to 65 years	12	Centre d’Etude des Cellules Souches	Allogenic	Monolayer	I/II	Monolayer implantation
3	ESC-RPE	NCT02903576	Stem Cell Therapy for Outer Retinal Degenerations	AMD; SMD; wet AMD	Completed	Brazil	18 to 90 years	15	Federal University of São Paulo	Allogenic	Suspension	I/II	Subretinal injection
4	ESC-RPE	NCT01469832	Safety and Tolerability of Sub-retinal Transplantation of Human Embryonic Stem Cell-derived Retinal Pigmented Epithelial (hESC-RPE) in Patients with Stargardt’s Macular Dystrophy (SMD)	SMD	Completed	UK	18 years and older	12	Astellas Institute for Regenerative Medicine	Allogenic	Suspension	I/II	Subretinal injection
5	ESC-RPE	NCT01344993	Safety and Tolerability of Sub-retinal Transplantation of hESC-derived RPE (MA09-hRPE) in Patients with Advanced Dry AMD	Dry AMD	Completed	US	55 years and older	13	Astellas Institute for Regenerative Medicine	Allogenic	Suspension	I/II	Subretinal injection
6	ESC-RPE	NCT01345006	Sub-retinal Transplantation of hESC-derived RPEs (MA09-hRPE) in Patients with Stargardt’s Macular Dystrophy	SMD	Completed	US	18 years and older	13	Astellas Institute for Regenerative Medicine	Allogenic	Suspension	I/II	Subretinal injection
7	iPSC-CEC	jRCTa031210199	iPSC -derived corneal endothelial cell substitutes for bullous keratopathy	Bullous keratopathy	Recruiting	Japan	45 to 85 years	3	Hirayama Masatoshi	Allogenic	Suspension	I	Subconjunctival injection
8	iPSC-RPE	jRCTa050210178	Clinical Research of allogeneic iPSC-RPE strip transplantation for RPE impaired disease	RPE-impaired disease	Recruiting	Japan	20 years and older	50	Kurimoto Yasuo	Allogenic	Cell strip	I/II	Subretinal transplantation
9	iPSC-RPE	UMIN000026003	A Study of transplantation of allogenic iPSC-derived RPE suspension in subjects with neovascular AMD	Wet AMD	Completed	Japan	50 to 85 years	5	Kobe City Eye Hospital	Allogenic	Suspension	I/II	Subretinal injection
10	iPSC-RPE	UMIN000011929	A Study of transplantation of autologous iPSC-derived RPE cell sheet in subjects with exudative AMD	Wet AMD	Completed	Japan	50 years and older	2	RIKEN	Autologous	Cell sheet	I	Subretinal transplantation
11	iPSC-RPE	NCT04339764	A Phase I/IIa Trial for Autologous Transplantation of iPSC-derived RPEs for Geographic Atrophy Associated with AMD	Dry AMD	Recruiting	US	55 years and older	20	National Eye Institute (NEI)	Autologous	RPE-plus-scaffold delivery	I/II	Subretinal transplantation

We herein enumerate the ongoing and closed clinical trials regarding PSC-derived RPE transplantation for the treatment of RPE diseases. We particularly address the origin of cell type (ESCs or iPSCs), the indication of transplantation, the delivered form (single-cell suspension, strip, or sheet) of stem cells, as well as the surgical approach to delivery. MD: macular degeneration; RP: retinitis pigmentosa; SMD: Stargardt’s macular dystrophy.
